# Buffalo Medical and Surgical Association

**Published:** 1882-08

**Authors:** Clayton M. Daniels

**Affiliations:** Secretary


### Buffalo Medical and Surgical Association.

*Special Meeting July 1st, 1882.*

President, Dr. A. R. Davidson, in the chair. Dr. C. M. Daniels, Secretary.

Minutes of last meeting read and approved.

Present—Drs. Rochester, Mynter, C. L. Dayton, Runner, Hopkins, Bartow, Barrett, Burghardt, Daggett, C. A. Ring, Hartwig, Bartlett, Howe, Warren, Moody, Fowler, Van Peyma, and by invitation, Drs. McBeth, Waldurff, Berkes, Mann, Coakley, Ingraham, Fell, and Frederick, also, Mr. A. Briggs and Mr. D. C. Beard.

Application for membership received from Dr. Coakley, who was duly elected.

*Essay*—By Dr. A. R. Davidson, subject: "Sewer Gas, and its Dangers." A full report of this paper is given in the July number of the Buffalo Medical Journal.

*Discussion*—Dr. Rochester expressed his thanks to the essayist for his excellent paper, in which he had made clear many points connected with house drainage, which were not generally understood, especially that of the syphonages of traps. He had himself given the subject a good deal of attention, but Dr. Davidson, by means of the ingenious apparatus with which he had demonstrated the practical working of an unventilated system of house drainage, had shown beyond question the uselessness of the ordinary trap without a ventilating attachment. The plan, which the lecturer proposed to prevent the entrance of sewer air into our houses, seemed to be a simple and practical one, and would, no doubt, avert the danger arising from this source. Since the last meeting of the Association, he had changed his views regarding the non-infecting qualities of "sewer gas"; now believing that it was often the cause of disease. He related to a case, where sewer had burst beneath a house, where it was covered with earth to a depth of four feet—there being no cellar—and where the "sewer air," (as he preferred to call it) penetrated upward into the house. He thought that the infecting property were similar to that of water infected with the germs of typhoid fever.

Dr. Coakley had listened to the paper with much interest, but thought that the plan, as portrayed upon the blackboard, was open to the objection, that the soil pipe was too frequently tapped by the pipes from other fixtures. He referred to his own experiences in building, and in his opinion all pipes should go to the basement, there uniting in a hopper, which should be connected to sewer by only one opening. The hopper first receiving all sewage from the house, and this hopper protected by a bell-shaped hood and connected by a pipe to a flue in a chimney.

Dr. Bartlett thought the subject of great importance, but would lay greater stress upon the necessity of ventilation outside of buildings, and hoped it would not be long before the sewers were ventilated by man-holes, etc.

Dr. Hopkins thanked the essayist for the able manner in which he treated the subject, and spoke of the sensational manner in which the dangers, arising from sewer air, were discussed and often magnified. He denied that it was possible to have spontaneous development of disease germs from the sewer air itself. He referred to the too often found illy-ventilated beds and bed-rooms.

Dr. Fell related a case, where a servant died of typhoid fever that seemed to have been caused by an abandoned and leaking sewer, running under the house.

Dr. C. L. Dayton appreciated the able manner in which the essayist explained the best way to get rid of sewer gas, and thought the public should be educated up to it. He hoped that the proceedings of this meeting would be published and the public thereby enlightened.

Dr. Davidson differed with Dr. Rochester regarding the possibility of the sewer gas itself having infecting properties, and objected to the plan of connecting ventilating pipes with the chimney flue.

*Voluntary Communication*—Dr. Mynter related a case of a man, 56 years of age, temperate, etc.; had soreness of heel, caused by being slightly punctured by a nail and which caused only a local inflammation. This, however, increased; foot became œdematous, and four free incisions were made. There was no bleeding; tension being so great that arterial circulation was cut off. Amputation was advised. The patient asked delay for two weeks, when hemorrhage occurred; amputated at middle of leg; arteries found atheromatous and filled with calcareous matter. Patient died on fifth day afterward. The case was of interest, as being one where the condition was not caused by alcoholism. He attributed it to the same causes as produced senile gangrene.

Dr. Rochester related similar cases and thought that age did not decide it, but that the cause was due to embolism, also, that they resembled phlegmonous erysipelas.

Dr. Hartwig related a case of spontaneous expulsion of fœtus, at fourth months, with membranes intact, and asked if any physician present had similar experience.

Dr. Bartlett said he had seen several from third to fifth month, and recounted one where a case had gone on to seventh month, when expulsion of fœtus, membranes and placenta took place. The fœtus had evidently been dead about three months, as cord was firmly around the neck and strangulation was complete; the size of the neck being compared to a thread.

On motion the reports as to prevailing diseases and other business was laid over to the next meeting.

The President appointed as a Finance Committee for the current year Drs. Hopkins, Mynter and Daniels.

The Association then adjourned to the first Monday in August.

CLAYTON M. DANIELS, *Secretary.*

